# Growth rate-dependent flexural rigidity of microtubules influences pattern formation in collective motion

**DOI:** 10.1186/s12951-021-00960-y

**Published:** 2021-07-19

**Authors:** Hang Zhou, Naoto Isozaki, Kazuya Fujimoto, Ryuji Yokokawa

**Affiliations:** Department of Micro Engineering, Kyoto University, Kyoto Daigaku-Katsura, Nishikyo-ku, Kyoto, 615-8540 Japan

**Keywords:** Microtubule, Flexural rigidity, Localization precision, Growth rate, Collective motion

## Abstract

**Background:**

Microtubules (MTs) are highly dynamic tubular cytoskeleton filaments that are essential for cellular morphology and intracellular transport. In vivo, the flexural rigidity of MTs can be dynamically regulated depending on their intracellular function. In the in vitro reconstructed MT-motor system, flexural rigidity affects MT gliding behaviors and trajectories. Despite the importance of flexural rigidity for both biological functions and in vitro applications, there is no clear interpretation of the regulation of MT flexural rigidity, and the results of many studies are contradictory. These discrepancies impede our understanding of the regulation of MT flexural rigidity, thereby challenging its precise manipulation.

**Results:**

Here, plausible explanations for these discrepancies are provided and a new method to evaluate the MT rigidity is developed. Moreover, a new relationship of the dynamic and mechanic of MTs is revealed that MT flexural rigidity decreases through three phases with the growth rate increases, which offers a method of designing MT flexural rigidity by regulating its growth rate. To test the validity of this method, the gliding performances of MTs with different flexural rigidities polymerized at different growth rates are examined. The growth rate-dependent flexural rigidity of MTs is experimentally found to influence the pattern formation in collective motion using gliding motility assay, which is further validated using machine learning.

**Conclusion:**

Our study establishes a robust quantitative method for measurement and design of MT flexural rigidity to study its influences on MT gliding assays, collective motion, and other biological activities in vitro. The new relationship about the growth rate and rigidity of MTs updates current concepts on the dynamics and mechanics of MTs and provides comparable data for investigating the regulation mechanism of MT rigidity in vivo in the future.

**Graphic Abstract:**

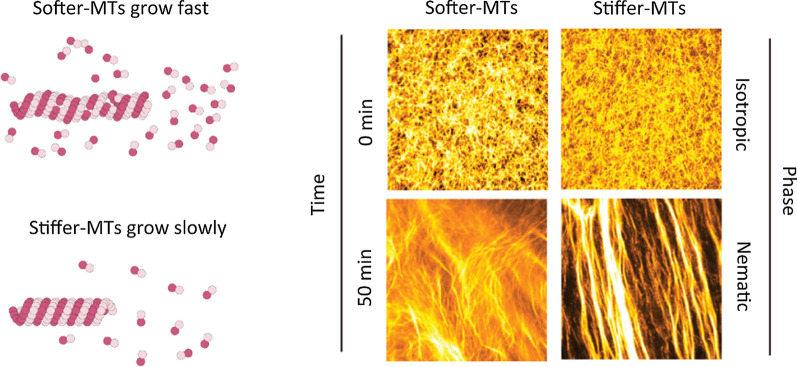

**Supplementary Information:**

The online version contains supplementary material available at 10.1186/s12951-021-00960-y.

## Introduction

Microtubule (MT) cytoskeletal filaments play an important role in supporting extended cellular structures, including axons and dendrites, because their flexural rigidity is 2–3 orders of magnitude higher than that of other cytoskeletal filaments, such as actin and intermediate filaments [1, 2]. In vivo, the mechanical stiffness of MTs can be dynamically controlled according to their intracellular function [3, 4]. In the in vitro reconstructed MT-motor systems, motor-driven MTs can be used as active matter in collective motion or transport of molecular cargoes as nanoscale shuttles or bio-robots [5–10]. As an intrinsic mechanical property of MTs, flexural rigidity determines the fluctuation of the free tip of MTs during the gliding assay and affects MT gliding behavior and orientation [10–14].

Owing to the crucial significance of MT rigidity in biological functions and engineering applications, numerous measurements on flexural rigidity or persistence length of MTs have been reported using a variety of measurement methods [14–39], such as thermal fluctuations [13, 17, 19, 20, 22, 26, 32], optical tweezers [18, 23, 28, 38], and atomic force microscopy [29]. From these studies, it was found that MT flexural rigidity can be modified by changes in the polymerization conditions, presence of MT-associated proteins (MAPs), and post-translational modification state of the tubulin dimer [40]. However, the reported values of flexural rigidity vary substantially between several studies, as listed in Additional file 1: Table S1. For example, the flexural rigidity of MTs that are polymerized in the presence of guanosine-triphosphate (GTP) and stabilized by paclitaxel, have been reported to vary between 0.03 and 3.2 × 10^−23^ N·m^2^—two orders of magnitude in range [14–19]. These discrepancies hamper our understanding of the regulatory mechanisms in MT rigidity, thereby challenging the precise manipulation or regulation of MT rigidity and impeding further investigation on the role of flexural rigidity on routine activities, including gliding motility and collective motion.

To address this issue, we developed a new method to evaluate the MT rigidity by improving the conventional measurement methods from two aspects. One is the localization precision of the shape of MTs during image analysis. Most previous studies measured MT flexural rigidity based on the deformation generated by an external force [17–25]. The deformation was measured by skeletonizing the MTs captured in fluorescence or differential interference contrast images and determining their positional shifts. The precision of the skeletonization method varies from 0.02 to 1 pixel, depending on the method. Lower precisions cause a larger measurement error in MT deformation. The other aspect is the variable parameters of polymerization that are not reported in published papers, such as the growth rate of the filaments. As the growth rate is sensitive to experimental conditions such as tubulin concentration, protein source, polymerizing temperature, and the binding of MAPs [41–48], the variation in growth rate causes inconsistency in the measured flexural rigidity.

To demonstrate our method, we investigated the effects of localization precision and growth rate on flexural rigidity. We evaluated MT flexural rigidity at low-level and nanometer-level localization precision using simulation analysis. Further, we modified the growth rate of MTs by altering tubulin concentration and measured flexural rigidity using nanometer-level localization precision. A new relationship between the growth rate and flexural rigidity of MTs was revealed that MT rigidity decreases and goes through three regimes with growth rate increases, which enables us to develop a potential quantitative method for designing the flexural rigidity of MTs.

To test the feasibility of this method, we chose the gliding motility assay as a platform to observe how growth rate-dependent flexural rigidity influences the collective motion of MTs, as flexural rigidity has been proven to influence the gliding behaviors and trajectories of MTs [10, 12–14]. Furthermore, we utilized machine learning to recognize, categorize, and verify the distinctive patterns of *softer MTs* and *stiffer MTs*.

Our study established a more accurate method of measuring MT rigidity by highlighting the importance of localization precision and growth rate in the measurement process. Furthermore, we developed a standard quantitative method to study the influences of MT rigidity on their routine behaviors by adjusting the growth rate of MTs.

## Materials and methods

### Reagent and protein preparations

All the reagents were purchased from Sigma-Aldrich, unless stated otherwise. Tubulin was purified from porcine brains after two cycles of polymerization and depolymerization [49], resulting in a final tubulin concentration of 282.7 µM. Recycled tubulin was prepared from tubulin by an additional cycle of polymerization and depolymerization to remove any non-polymerized tubulin. Fluorescently labeled tubulin was prepared by adding 50 M or 15 M excess succinimidyl ester-conjugated tetramethylrhodamine (C-1171, Invitrogen, USA) or Alexa Fluor™ 488 (A-20000, Invitrogen), respectively, to tubulin. His6-tagged kinesin-1 (1 ~ 465aa) was purified from *Escherichia coli* using a nickel-nitrilotriacetic acid affinity resin according to a previously described method [50]. The prepared proteins were stored in liquid nitrogen until use.

### MT fluctuation model

We developed a model using our previous method to simulate the thermal fluctuations of MTs with specific length (*L*) and defined flexural rigidity (${\kappa }_{set}$) [10]. MTs that were assumed to be tethered at one end behaved as a cantilever beam in this model. The immobilized end was set as the origin of the orthogonal coordinate system. The shape of the MT-free segment, *y*(*s*), was expressed by the superposition of sine, cosine, hyperbolic sine, and hyperbolic cosine waves, *W*_*n*_(*s/L*) [51].

1 $$y(s)=\sum\limits_{n=1}^{\infty }\sqrt{\frac{1}{L}} {a}_{n}{W}_{n}(\frac{s}{L})$$

and

2 $${W}_{n}\left(\frac{s}{L}\right)=\frac{-\text{cosh}{(q}_{n})-\text{cos}({q}_{n})}{\text{sin}\left({q}_{n}\right)+\text{sinh}\left({q}_{n}\right)}\left(\text{sin}\left(\frac{{q}_{n}s}{L}\right)-\text{sinh}\left(\frac{{q}_{n}s}{L}\right)\right)+\text{cos}\left(\frac{{q}_{n}s}{L}\right)-\text{cosh}\left(\frac{{q}_{n}s}{L}\right)$$

where, *s* is the path length from the tethered end along the MT, and *q*_*n*_ is 1.875 (*n* = 1), 4.695 (*n* = 2), 7.855 (*n* = 3), and (*n* − 0.5)*π* (*n* ≥ 4) [51].

Equating thermal energy with MT bending energy calculated from MT deformation enables us to derive MT flexural rigidity, *κ*, with the variance of amplitude in each *n*th mode, *var*(*a*_*n*_) [52].

3 $$\kappa =\frac{{k}_{B}T}{\mathit{var}({a}_{n})}{\left(\frac{L}{{q}_{n}}\right)}^{4}$$

Here, *k*_B_ is Boltzmann’s constant, *T* is the absolute temperature under experimental conditions (298 K), and *a*_*n*_ is the *n*th mode amplitude, which follows a Gaussian distribution with a mean of zero and variance of *var*(*a*_*n*_). The thermal fluctuation model of MTs was generated by setting *L* to 5, 10, and 30 µm, and *κ*_*set*_ to 0.03, 0.3, and 3 × 10^−23^ N m^2^, within the range of previously measured rigidities for GTP-polymerized, paclitaxel-stabilized MTs [14–19].

The measured flexural rigidity, *κ*_meas_, was calculated from the rounded coordinates at different localization precisions. First, the *xy*-coordinates of the shape of MTs in the model were rounded to the nearest multiple of the given localization precision: 1 nm (≈ 0.01 pixels), 10 nm (≈ 0.1 pixels), and 100 nm (≈ 1 pixel). For example, *y* = 236.8 nm was converted to *y* = 237 nm under a localization precision of 1 nm, *y* = 240 nm under 10 nm, and *y* = 200 nm under 100 nm. Thereafter, MT was divided into *N* + 1 points with the converted coordinates of (*x*′_*k*_, *y*′_*k*_). The *n*^th^ mode amplitude of the converted shape, *a′*_*n*_, was Fourier inverse transformed from Eq. (1) and solved using the midpoint method as follows:

4 $${a}_{n}^{\prime}\approx \sqrt{\frac{1}{{L}^{\prime}}}\sum_{k=1}^{N}{y}_{k}^{\prime}\Delta {s}_{k}^{\prime}{W}_{n}\left(\frac{{{s}_{k}^{\prime}}^{\text{mid}}}{{L}^{\prime}}\right), n=1, \dots ,N-1$$

where, *Δs*′_*k*_ is the length of the infinitesimal segment calculated by

5 $$\Delta s_{k}^{\prime}=\sqrt{{\left({x}_{k+1}^{\prime}-{x}_{k}^{\prime}\right)}^{2}+{\left({y}_{k+1}^{\prime}-{y}_{k}^{\prime}\right)}^{2}}$$

6 $$L^{\prime}=\sum_{k=1}^{N}\Delta {s}_{k}^{\prime}
$$

and

7 $${{s}_{k}^{\prime}}^{\text{mid}}=\Delta s_{1}^{\prime}+\Delta s_{2}^{\prime}+\Delta s_{3}^{\prime}+\cdots +\Delta s_{k-1}^{\prime}+\frac{1}{2}\Delta s_{k}^{\prime}$$

The *κ*_*meas*_ was measured under each localization precision by substituting *a*′_*n*_ and *L*′ into Eq. (3).

### Measurement of flexural rigidity

Seed MTs were polymerized by incubating Alexa Fluor™ 488-labeled tubulin and recycled tubulin at a 1:2 molar ratio in the presence of 1 mM DTT and 1 mM GMPCPP at 37 °C for 30 min. The seed MTs were biotinylated by incubating with 20 M excess succinimidyl ester-conjugated biotin (B1606, Invitrogen) at 37 °C for 30 min and quenched with 200 M excess potassium glutamate at 37 °C for 10 min. The partially biotinylated MTs were prepared by incubating the biotinylated seeds in the tubulin solution at various concentrations (20, 30, 35, 40, 50, 75, 100, 150, and 200 μM). All MTs were stabilized with 20 µM paclitaxel after elongation.

**Fig. 1:**
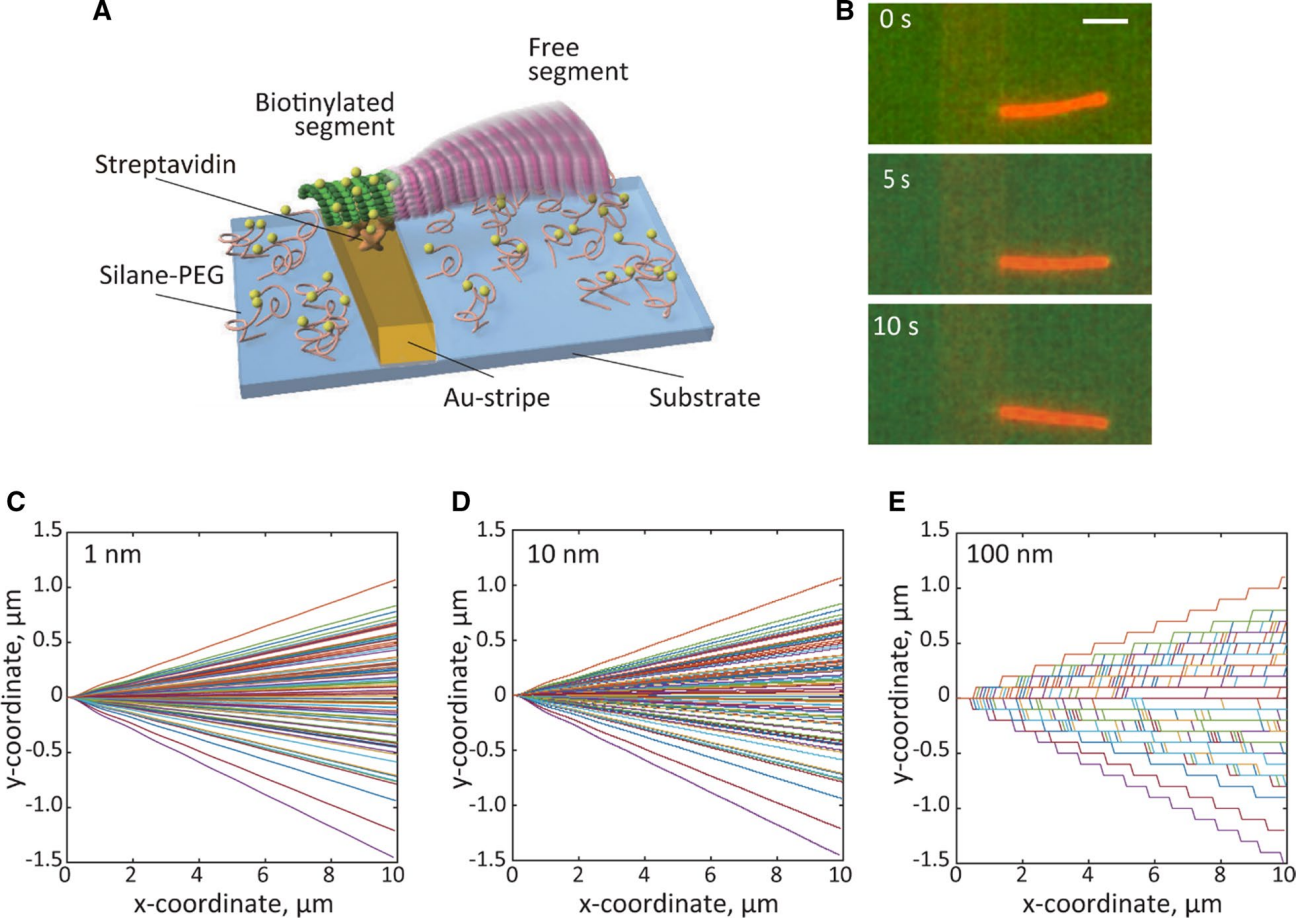
Flexural rigidity measurement of MTs. **A** Illustration of a partially biotinylated MT, that was immobilized onto the Au-stripe-patterned substrate with biotin–streptavidin bindings and the free segment fluctuated as a cantilever beam under Brownian motion. **B** Sequential images of a fluctuating MT (orange red) immobilized on the Au-stripe (light orange). Scale bar = 5 μm. Theoretical model of MT thermal fluctuation with localization precision of **C** 1 nm, **D** 10 nm, and **E** 100 nm. MT shapes in 500 frames are superimposed with different colors

Flow chambers were constructed by bonding the Au-stripe-patterned SiO_2_ substrate (see Additional file 1: Figure S1 for fabrication details) and a coverslip (C218181, Matsunami Glass, Japan) with 10-µm thick double-sided tapes (7070W, Teraoka Seisakusho, Japan). We introduced 2 mg mL^−1^ streptavidin and incubated thrice for 3 min for non-specific binding on the Au-patterned substrates. After washing the flow chamber with BRB80 (80 mM PIPES, 1 mM EGTA, and 1 mM MgCl_2_), the partially biotinylated MTs were immobilized via biotin–streptavidin binding by incubation for 5 min (Fig. 1A). Free MTs were washed with BRB80 containing an O_2_ scavenger system (8.0 µg mL^−1^ catalase, 25 mM d-glucose, 20 µg mL^−1^ glucose oxidase, 1% β-mercaptoethanol, 20 mM DTT, and 20 µM paclitaxel). The flow chamber was then sealed with clear nail polish to prevent drying or leakage.

MTs were observed under an IX73 inverted epifluorescence microscope (Olympus, Japan) with an excitation filter (GFP/DsRed-A-OMF, Opto-Line International, Inc., USA), a complementary metal–oxide–semiconductor camera (ORCA-Flash 4.0 V2, Hamamatsu Photonics, Japan), image-splitting optics (W-VIEW GEMINI, Hamamatsu Photonics) with a bandpass emitter (FF01-512/25-25 and FF01-630/92-25, Semrock, USA), dichroic mirror (FF560-FDi01-25 × 36, Semrock), and oil-immersion objectives. The magnification and exposure time were set to 100 × (NA 1.4) and 100 ms, respectively. To observe MT thermal fluctuation, the frame rate and recording period were set to 2.5 frames s^−1^ and 200 s, respectively. We used an ND25 filter equipped with a shutter (VMM-D3, Uniblitz, USA). Optical images were stored as sequential image files in TIFF format using the HCImage software (Hamamatsu Photonics) (Fig. 1B and Additional file 2: Movie S1, Additional file 3: Movie S2, Additional file 4: Movie S3, Additional file 5: Movie S4). The MT shapes were determined using the MT tracking software, fluorescence image evaluation software for tracking and analysis (FIESTA) [53] with sub-pixel resolution (0.02 pixels, ~ 2.2 nm) via 2D Gaussian fitting (Additional file 1: Figure S2).

Multiple significance tests were performed on all data using Steel–Dwass tests at a critical value of *p* < 0.05, and normality or log-normality was tested among outlier-removed data using Lilliefors tests at a critical value of *p* > 0.05. All curve fittings were performed using the least-squares method in MATLAB (R2019b).

### Growth rate measurement using TIRF microscopy

A flow chamber was constructed by bonding two coverslips (C218181 and C024361, Matsunami Glass) with 50-µm thick double-sided tapes (400P50, Kyodo Giken Chemical, Japan). First, 50 μg mL^−1^ neutravidin was introduced and incubated for 5 min at 25 °C. After washing the channel thoroughly with BRB80, 1% Pluronic solution was introduced into the flow cell for 5 min at 25 °C and washed twice with BRB80.

The partially biotinylated seed MTs were introduced and selectively immobilized onto substrates via biotin–neutravidin binding with a 5 min incubation period. After washing the free seed MTs, a mixture of non-labeled and tetramethylrhodamine-labeled tubulin, MgSO_4_, and GTP was introduced into the flow cell. The final tubulin concentration was modified to 20, 30, 35, 40, 50, 75, 100, 150, and 200 μM, similar to the flexural rigidity measurements. The flow cell was sealed with clear nail polish and placed on a 37 °C heater installed on the stage of the microscope.

An EMCCD camera (iXon Ultra 897, Andor Technology, UK) and an inverted microscope (IX 71, Olympus) were used for fluorescence observation of the MT elongation process. Laser light sources (DPGL-2050F, Suwtech, China) with a wavelength of 532 nm were used as the excitation light source. The laser beam path was adjusted such that the excitation light was in the total reflection state. A fluorescent mirror unit (U-MWIG 3, Olympus) was used to separate the excitation light and fluorescence. To measure the elongation rate, 500 fluorescence images were captured with an exposure time of 100 ms, binning 1 × 1, using a 60 × objective lens (APON 60 XOTIRF, Olympus) for total internal reflection fluorescence (TIRF) microscopy. Image data were acquired in the TIFF format using the image acquisition software (Andor iQ 3, Andor). From the obtained sequential fluorescence images, the elongated lengths of the MTs were measured and the elongation rates were calculated using ImageJ (National Institutes of Health). Multiple significance tests were performed on all data using Steel–Dwass tests with a critical value of *p* < 0.05.

### In vitro gliding assay

The coverslips (C218181 and C024361, Matsunami Glass) used for the gliding assay were treated with piranha solution at 60 °C for 10 min, rinsed with deionized water and then dried with nitrogen gas. The central area (5 × 5 mm) of the coverslip was exposed to plasma with the surrounding area covered by tape. Two PIPES-based buffer solutions were used in the assay: (i) casein buffer, with 0.3 mg mL^−1^ casein dissolved in the BRB80 and (ii) motility buffer, BRB80 with 5 mM ATP, 0.3 wt% methylcellulose, and O_2_ scavenger system.

The gliding assay was conducted by introducing MTs onto kinesin molecules with motility buffer (Fig. 5A). First, 2 mg mL^−1^ streptavidin was introduced and incubated for non-specific binding to the substrate. After washing the chamber with BRB80 solution, biotin-conjugated kinesin-1 (0.154 mg mL^−1^) was added and incubated for 5 min and immobilized on the glass via biotin–avidin binding. After washing with casein buffer, MTs and motility buffer were introduced into the chamber. All gliding assays were performed at 25 °C. The gliding motion assays were observed using an inverted epifluorescence microscope (IX73, Olympus) with an excitation filter (GFP/DsRed-A-OMF, Opto-Line International, Inc.) and 60 × oil-immersion objectives. The exposure time was set at 50 ms with a frame rate of 1 frame s^−1^ and a recording period of 300 s. The addition time of the motility buffer was set as 0 s, and the whole observation time for each assay was 60 min. The captured images were stored as sequential image files in TIFF format using the HCImage software (Hamamatsu Photonics).

### MT pattern analysis using machine learning

A deep convolutional neural network (CNN) model including pre-trained ResNet50 top layers, a global average pooling layer, and a final dense layer activated using the Softmax function was constructed to classify the two MTs (Fig. 5B) [54]. We captured 1400 MT pattern images and 700 images each from *softer-MT* and *stiffer-MT* groups from 10 independent experiments for the dataset (840 images for the training process, 360 images for the validation process, and 200 images for the test process). All the images were cropped and resized to 224 × 224 pixels as input images for the model. The ground truth of the images was manually labeled. The model was trained by monitoring the validation loss, and the parameters were updated using the Adam optimizer. The model was trained for 20 epochs with a batch size of 32, and a final validation accuracy of 98% was obtained (Additional file 1: Figure S5). The calculated probabilities for each class were used for MT classification, and the sum of the probabilities was 100%. Tensorflow2.0 (www.tensorflow.org) and Keras API (keras.io) were used during the entire process.

A score class activation map (Score-CAM) was introduced to visually interpret the prediction strategy of the CNN classifier by obtaining the weights of each activation map on the predicted class [55]. For the deep learning model *f* (), we used input *X* and output *Y*: *Y* = *f* (*X*).

The activation of the input *X*_0_ generated in the convolution layer *l* is indicated as ${A}_{l}$, and the *k*th channel activation of ${A}_{l}$ is ${A}_{l}^{k}$. Further, ${A}_{l}^{k}$ is extracted, normalized, and upsampled to an input size of ${M}_{l}^{k}$. ${M}_{l}^{k}$ was used as a mask to be projected onto the original image ${X}_{0}$. For a given baseline ${X}_{b}$, the contribution ${S}_{k}^{c}$ of ${A}_{l}^{k}$ to the output class *c* is calculated as follows [55]:

8 $${S}_{k}^{c}={f}^{c}({M}_{l}^{k}\circ {X}_{0})-{f}^{c}({X}_{b})$$

where, $\circ$ indicates the element-wise product and ${\alpha }_{k}^{c}$ is the corresponding weight of ${A}_{l}^{k}$ to the target class *c*.

9 $${\alpha }_{k}^{c}={e}{x}{p}({S}_{k}^{c})/\sum_{k}{e}{x}{p}({S}_{k}^{c})$$

The heatmap ${L}_{{s}{c}{o}{r}{e}-{C}{A}{M}}^{c}$ is generated by the linear combination of activation maps ${A}_{l}^{k}$ and their weights ${\alpha }_{k}^{c}$ on target class *c*.

10 $${L}_{{s}{c}{o}{r}{e}-{C}{A}{M}}^{c}={R}{e}{L}{U}\left(\sum_{k}{\alpha}_{k}^{c}{A}_{l}^{k}\right)$$

Here, the activation function ${R}{e}{L}{U}$ was used to select the features with positive importance scores.

## Results and discussion

### Flexural rigidity of MTs under different localization precisions

The flexural rigidity of MTs was measured from the rounded coordinates at different localization precisions (Fig. 1). Figure 1C–E show the converted shapes of MTs under the localization precision of 1, 10, and 100 nm with MT length *L* = 10 µm and defined flexural rigidity *κ*_set_ = 0.3 × 10^−23^ N m^2^. The shapes in 500 frames were superimposed with different colors. Under low localization precision, the rounded MT shapes were discontinuous and substantially deviated from the original shapes.

**Fig. 2:**
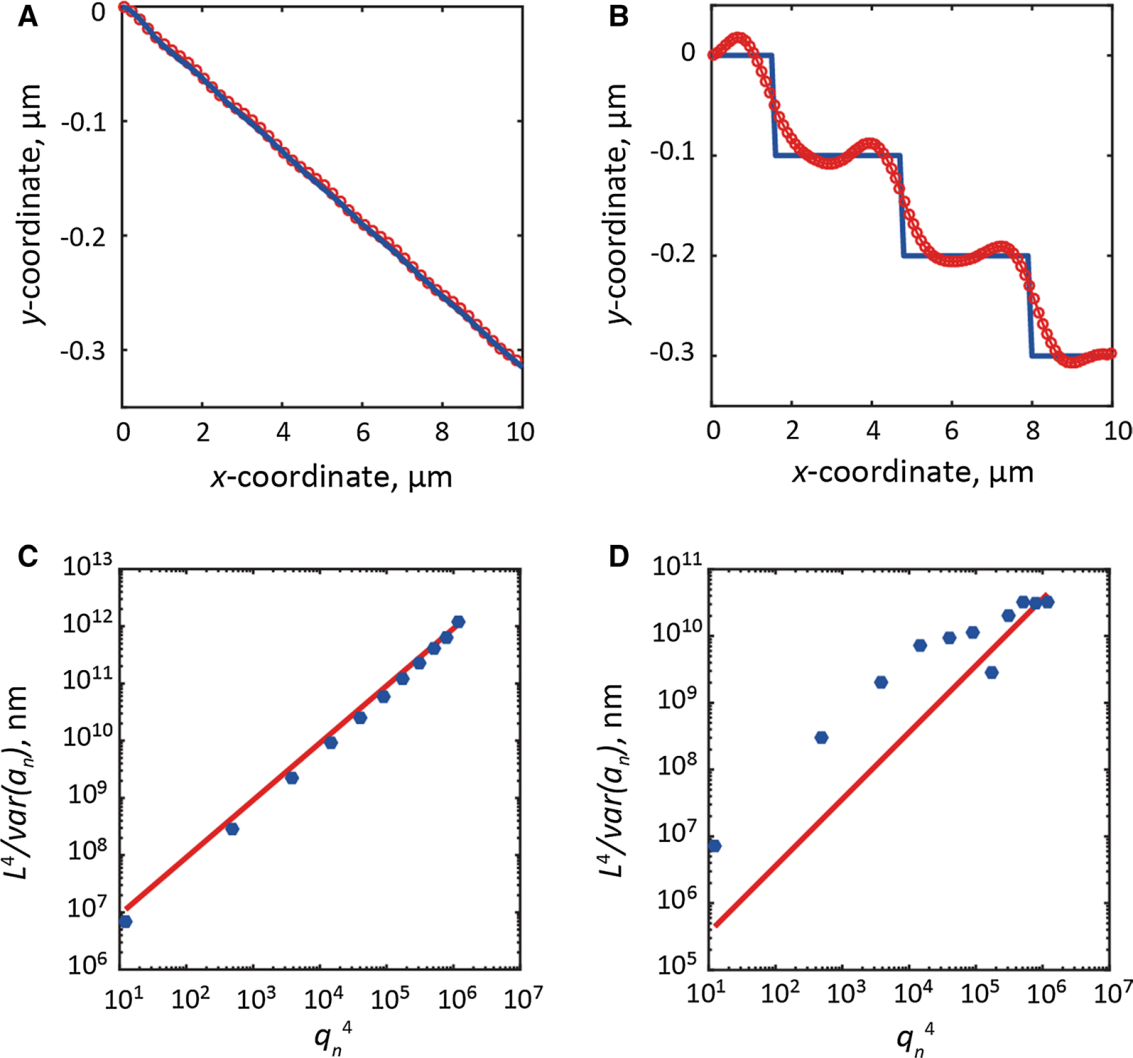
Measurement process of *κ* for an MT of *L* = 10 µm and *κ*_*se*t_ = 0.3 × 10^−23^ N·m^2^ with different localization precision. MT shapes (blue lines) and fitted curves (red lines and dots) with localization precision of **A** 1 nm and **B** 100 nm. Relationship between *q*_*n*_^*4*^ and *L*^*4*^/*var*(*a*_*n*_) with localization precision of **C** 1 nm and **D** 100 nm. Blue dots: measurement results. Red lines: fitted straight lines

The shape and approximate value of *κ* for an MT of *L* = 10 µm and *κ*_*se*t_ = 0.3 × 10^−23^ N m^2^ was calculated using Eq. (1), as shown in Fig. 2A, B. The shapes of MTs are represented by the blue line, whereas the fitted curves are indicated by the red lines and red dots with localization precision of (A) 1 nm and (B) 100 nm. As the localization precision decreases, the thermal fluctuation of MTs cannot be traced precisely, and the accuracy of the approximation drops remarkably. The discontinuous shape under 100 nm localization precision cannot be represented well by Eq. (1), thereby leading to a significantly larger fitting error than that under 1 nm localization precision. Given that *k*_B_*T* is constant at a specific temperature, the flexural rigidity, *κ*, can be derived from the slope between *q*_n_^4^ and *L*^4^/var (*a*_n_) through Eq. (3) [52]. Figure 2C and D show the relationship between *q*_n_^4^ and *L*^4^/var (*a*_n_) with localization precision of (C) 1 nm and (D) 100 nm on a logarithmic graph. When the localization precision is high, *q*_n_^4^ and *L*^4^/var (*a*_n_) are linearly related regardless of the number of modes (Fig. 2C). However, when the localization precision is low, *q*_n_^4^ and *L*^4^/var (*a*_n_) maintain a linear relationship only in the low-order mode (n ≤ 4) (Fig. 2D). This is because thermal fluctuation cannot be precisely tracked in the higher-order (*q*_n_) mode, in which the deformation of the shape of MTs is smaller than the localization precision.

### Nanometre-level localization precision eliminates the measurement error

**Fig. 3:**
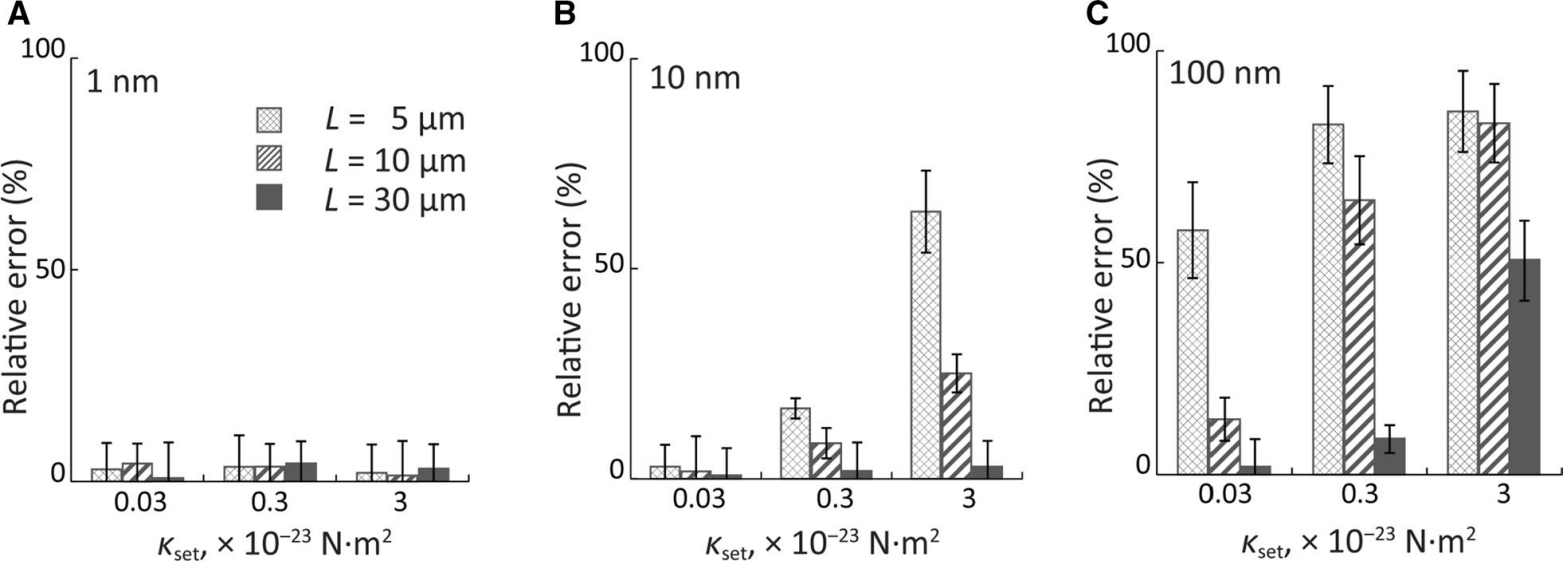
Error measurement of MT flexural rigidity under each condition of *L*, *κ*_set_, and localization precision of **A** 1 nm, **B** 10 nm, and **C** 100 nm. Cross-hatched bars: *L* = 5 μm. Hatched bars: *L* = 10 μm. Shaded bars: *L* = 30 μm

The relative measurement error of *κ* due to the digitization of the shape of MTs was determined from 1–*κ*_meas_/*κ*_set_. Figure 3 shows the relative measurement error of flexural rigidity under each condition of *L* (5, 10, and 30 μm), *κ*_set_ (0.03, 0.3, 3 × 10^−23^ N·m^2^), and localization precision of (A) 1 nm, (B) 10 nm, and (C) 100 nm. The measurement error was found to be affected by the localization precision, MT length, *L,* and the defined flexural rigidity, *κ*_set_. Lower localization precision, smaller contour lengths, and larger *κ*_set_ will all result in larger measurement errors. Under low localization precision (10 and 100 nm), the flexural rigidity is largely underestimated, especially for short and/or stiff MTs, by overestimating thermal fluctuations.

These results help explain previous discrepancies reported in the literature. For instance, Venier et al. [24] reported a three-fold smaller *κ* than Valdman et al. [35]. Both studies adopted almost the same conditions (tubulin concentration, nucleotide, MT length, and paclitaxel stabilization) but different localization precision for determining MT shape (1 pixel for Venier et al. [24] and 0.1 pixels for Valdman et al*.* [35]). As indicated in Fig. 3C, the pixel-level localization precision of Venier et al*.* [24] may have caused the underestimation of *κ*. The same can be said for the difference in reported *κ* between Hawkins et al. [21] and Lopez et al. [36]. Although most of the conditions were the same, Hawkins et al. [21] skeletonized the MT image with 1-pixel localization precision and reported a 3.5-fold smaller *κ* than Lopez et al*.* [36] with 0.1-pixel localization precision.

Our previous studies have shown that MT flexural rigidity is independent of *L* [10]; however, the current results on simulated filaments indicate that the contour length, *L,* influences the measurement accuracy of flexural rigidity. Venier et al. [24] and Mickey and Howard [17] used similar polymerization conditions and pixel-level localization precision, but different lengths of the MTs in each study. Venier et al. [24] analyzed shorter MTs (8–15 µm) than Mickey and Howard [17] (24–68 µm) and obtained a 2.8-fold smaller *κ*. This is possible because the small deflections of short MTs caused an underestimation of *κ* (Fig. 3C).

The results also indicate that the flexural rigidities of MTs with a large *κ* are more likely to be underestimated, especially at low localization precision. Mickey and Howard [17] reported that *κ* increased about 1.3 times with tau protein bound. Whereas, Felgner et al. [18] used a similar ratio of tau to tubulin and reported that the *κ* increased approximately 2.5 times when measured using optical tweezers. The discrepancy in the increase of *κ* between the two groups is probably caused by the underestimation of *κ* due to the low pixel-level localization precision used by Mickey and Howard [17]. In addition, Cassimeris et al. [19] co-polymerized XMAP215, another type of MAPs, with tubulin and did not find any change in *κ* under pixel-level localization precision. This could be caused due to a very low precision that hinders the detection of changes in *κ* because of the larger measurement error. Thus, it would be interesting to measure the effects of XMAP215 with improved localization precision.

It is noteworthy that, when the localization precision is at 1 nm, the relative measurement error is less than 5% regardless of the value of *L* or *κ*_set_ (Fig. 3A and Additional file 1: Figure S3A). Thus, the measurement error caused by *L* and *κ* can be significantly reduced by improving the localization precision. Therefore, nanometre-level localization precision is indispensable for the precise and accurate measurement of flexural rigidity for MTs of various lengths and stiffnesses. Further, by improving the localization precision to the nanometre level, it is possible to obtain new findings by reinvestigating the influences of these factors on *κ*.

### Dependence of MT flexural rigidity on the growth rate

**Fig. 4:**
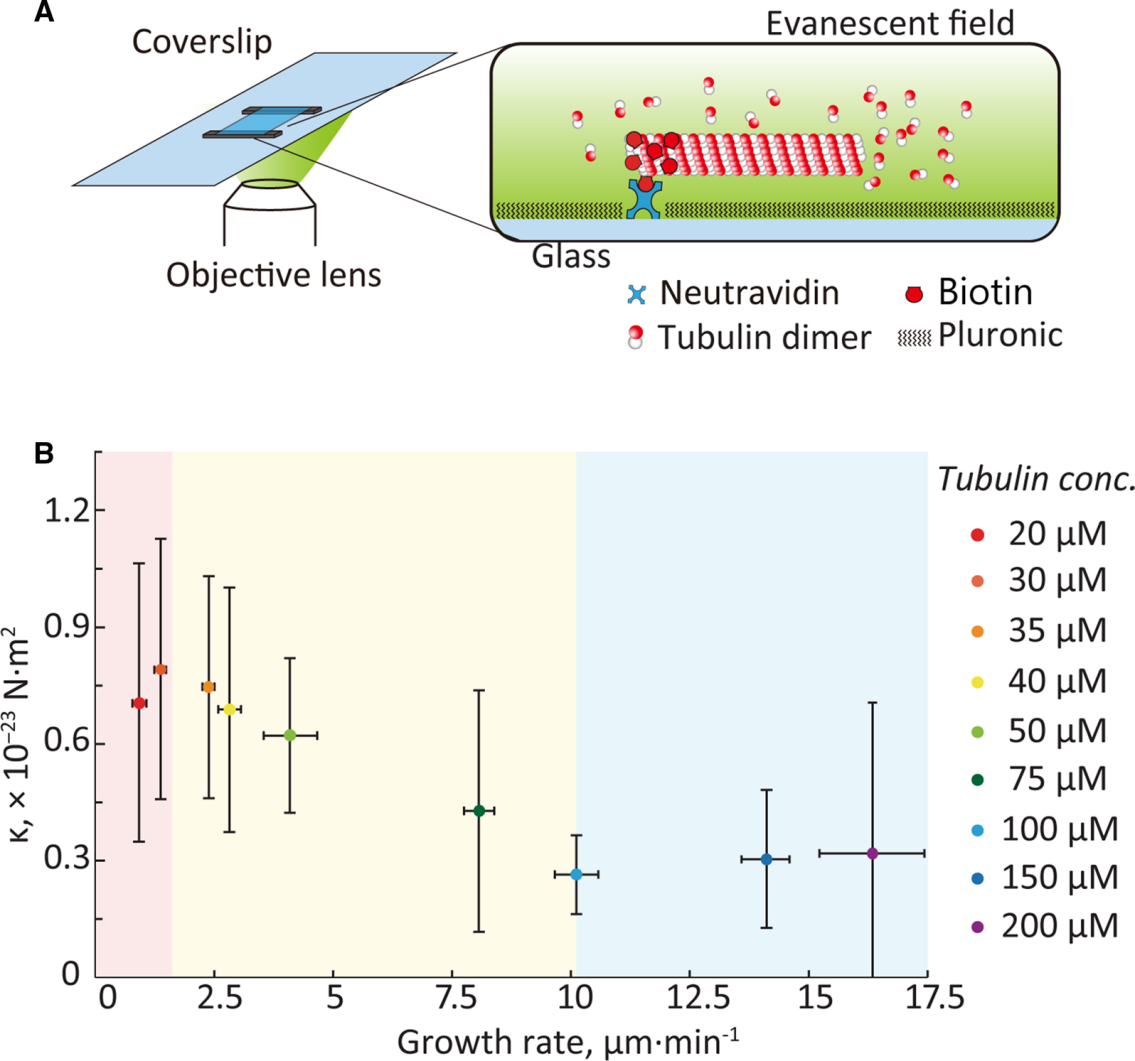
Relationship between growth rate and flexural rigidity of MTs. **A** Growth rate measurement of MTs elongating under different tubulin concentrations as observed using TIRF microscopy. The partially biotinylated seed MTs were immobilized onto substrates via biotin–neutravidin bindings. Tubulin protein solution with a specific concentration was introduced into the flow cell and MT elongation process was observed directly. **B** MT flexural rigidity decreases with an increase in growth rate, which follows a three-state change and is illustrated with red, yellow, and blue background. The corresponding tubulin concentrations of the three stages are ≤ 30 μM, 30–100 μM, and > 100 μM, respectively. MTs incubated in tubulin concentrations from 20–200 μM are illustrated by the dots with different colors

The measured values of flexural rigidity and growth rates of MTs polymerizing with tubulin concentrations of 20–200 µM are summarized in Additional file 1: Table S2. The relationship between the growth rate and flexural rigidity of the MT is shown in Fig. 4B. The growth rate was found to significantly increase with higher tubulin concentrations within the range of 30–150 µM. The slope of the growth rate was 0.1 µm min^−1^ µM^−1^, which is approximately tenfold faster at 150 µM than that at 30 µM. No significant differences in growth rates were observed between 20 µM and 30 µM as well as between 150 µM and 200 µM using the Steel–Dwass test (*p* > 0.05, Additional file 1: Table S3). Therefore, the whole variation process of growth rate could be divided into three phases according to their statistical differences (Additional file 1: Table S3), with tubulin concentration values of ≤ 30 μM, 30–100 μM, and > 100 μM, which are illustrated by a red, yellow, and blue background, respectively (Fig. 4B).

Meanwhile, flexural rigidity was found to be dependent on the growth rate. When the growth rate was less than ~ 1.4 μm min^−1^, that is, the tubulin concentration was lower than 30 μM, the flexural rigidity remained similar to that observed between 20 and 30 μM (no statistical significance of MT rigidity at 20 and 30 µM was detected, Additional file 1: Table S4). Once the MTs elongate faster than 1.4 μm min^−1^, the flexural rigidity tends to decrease as the growth rate increases. At a growth rate of ~ 10.1 µm min^−1^ (tubulin concentration higher than 100 μM), the flexural rigidity becomes independent of the growth rate.

The dependence of the flexural rigidity on the growth rate can be explained by the number of defects generated and repaired during polymerization. As reported by Schaedel et al*.* [56], faster growth tended to generate lattice defects on MTs, which reduced the MT flexural rigidity. We found that when the tubulin concentration was too low to facilitate fast MT polymerization (20–30 µM), MTs show high bending stiffness, most likely due to the formation of fewer lattice defects. At a low growth rate, MTs repair their tubulin defects while elongating, thereby resulting in higher flexural rigidity with fewer defects [56].

Fast growth of MTs can result in accumulation of defects that cannot be repaired. Thus, in this regime (30–100 μM), higher number of defects in MTs will result in softer filaments. MT rigidity was found to decline monotonically as the growth rate increased in the region (Fig. 4B), which is consistent with the results of Janson and Dogterom [25, 26].

As the tubulin concentration during polymerization was increased to over 100 µM, the growth rate increased and then reached the upper limit value (no significant differences in the growth rate at 150 and 200 µM were observed in Additional file 1: Table S3). Correspondingly, no change in rigidity was observed (Fig. 4B, no significant difference of MT rigidities at 100, 150, and 200 µM were detected in Additional file 1: Table S4), which indicates that there should be a certain upper limit number of lattice defects accumulating on MTs. Once the quantity of tubulin defects exceeds a certain threshold, fatal structural damages will occur to break MT or cause MT depolymerization. As a result, when MTs grow fast to exceed the upper threshold value (~ 10.1 µm min^−1^), the number of tubulin defects of the surviving MTs remains constant at the upper limit. Therefore, no additional tubulin defects would be generated, the flexural rigidity of MTs keeps at a constant low level with the tubulin concentration higher than 100 μM.

Results from previous studies display discrepancies in their reported rigidity values owing to the different polymerization speeds. For instance, Valdman et al. [35] and Lopez et al. [36] performed experiments using the same measurement and conditions (localization precision, nucleotide, MT length, and paclitaxel stabilization) except for tubulin concentration (Valdman et al. [35], 20 μM; Lopez et al. [36], 50 μM). These publications were from the same group, and therefore, there is a likelihood that the tubulin type was identical. However, the measured *κ* reported by Valdman et al. was approximately 1.4-fold larger than that reported by Lopez et al. Although the growth rates were not quantified or reported, it is conceivable that the growth rate observed by Valdman et al. [35] would be smaller than that observed by Lopez et al. [36] because of the difference in the tubulin concentrations. Therefore, we highly recommend future measurements of flexural rigidity to include a measurement of the growth rate for the tubulin polymerization methods used in this study.

Furthermore, our results reveal the relation between MT growth rate and rigidity with a wider range of tubulin concentration (20–200 µM) than that in previous studies (4.5–28 µM) [25, 26, 57]. For instance, the growth rate and flexural rigidity of MTs polymerizing with a higher tubulin concentration (> 100 µM, blue area in Fig. 4B) are 10.1–16.3 µm min^−1^ and ~ 0.3 × 10^−23^ N·m^2^ (Additional file 1: Table S2), respectively, which corroborate the reported data of MTs in vivo (growth rate of 12–24 µm min^−1^ and flexural rigidity of 0.3–0.4 × 10^−23^ N·m^2^) [58, 59]. Interestingly, although the reported tubulin concentration in cells (4–24 µM) is much lower than that used here (> 100 µM), growth rates of MTs in cells are still comparable with or even faster than that observed in our results [60, 61]. The effect of tubulin concentration on the growth of MTs could be compensated by various kinds of MAPs in cells, such as the plus-end tracking proteins, CLASP, CLIP-170, XMAP215, and EB1 [4, 62]. However, the influences of these MAPs on MT rigidity are still unclear. Combined with our results, further experiments on the relationship between growth rate and rigidity in the presence of MAPs will induce an agreement in understanding the factors that influence rigidity in vivo.

Moreover, our results provide a quantitative link between growth rate and flexural rigidity for bare MTs, which suggests a potential method for designing flexural rigidity of MTs in vitro. Flexural rigidity is an important parameter that affects the direction of MT gliding. MTs modified with different flexural rigidities have been widely applied in nano- and bioengineering applications, including molecular shuttles, active self-organization, and collective motion [8–10]. Although the flexural rigidity of MTs can be modified by various MAPs, nucleotides, and MT-stabilizing reagents [16–21, 25–27, 36], they are also known to influence the molecular structure and biophysical functions of MTs [4, 36, 62, 63]. For instance, Janson and Dogterom [25, 26] polymerized MTs with/without oxygen-scavenging system (4 mM dithiothreitol, 0.2 mg ml^−1^ catalase, 0.4 mg ml^−1^ glucose oxidase, and 50 mM glucose) and found the oxygen-scavenger may promote the polymerization speed of MTs from 1.5 to 2.72 µm min^−1^ and soften MTs from 2.73 to 1.74 × 10^−23^ N·m^2^ (Additional file 1: Table S1). Their studies suggested that regulating growth rate could become a potential method to control MT rigidity, however, no specific implementable scheme to realize this idea so far. Here, the newfound relationship of growth rate and MT rigidity controlled by tubulin concentration addressed this issue. Compared with these conventional modification methods [16–21, 25–27, 36], our growth rate-dependent method is controlled solely by the concentration of tubulin. Therefore, the method not only simplifies the preparation of MTs, but also avoids uncertain influences on MTs, which will widen the applicability of the flexural rigidity for engineering applications.

### Growth rate-dependent flexural rigidity influences the collective motion in the MT motility assay

Two groups of MTs were used to perform the MT gliding assays. *Softer-MTs* were polymerized at 100 μM tubulin concentration with the growth rate of 10.10 ± 0.46 μm min^−1^ and *κ* of 0.27 ± 0.10 × 10^−23^ N·m^2^, whereas *stiffer-MTs* were polymerized at 30 μM tubulin concentration with the growth rate of 1.37 ± 0.07 μm min^−1^ and *κ* of 0.80 ± 0.34 × 10^−23^ N·m^2^ (Fig. 4B and Additional file 1: Table S2).

**Fig. 5:**
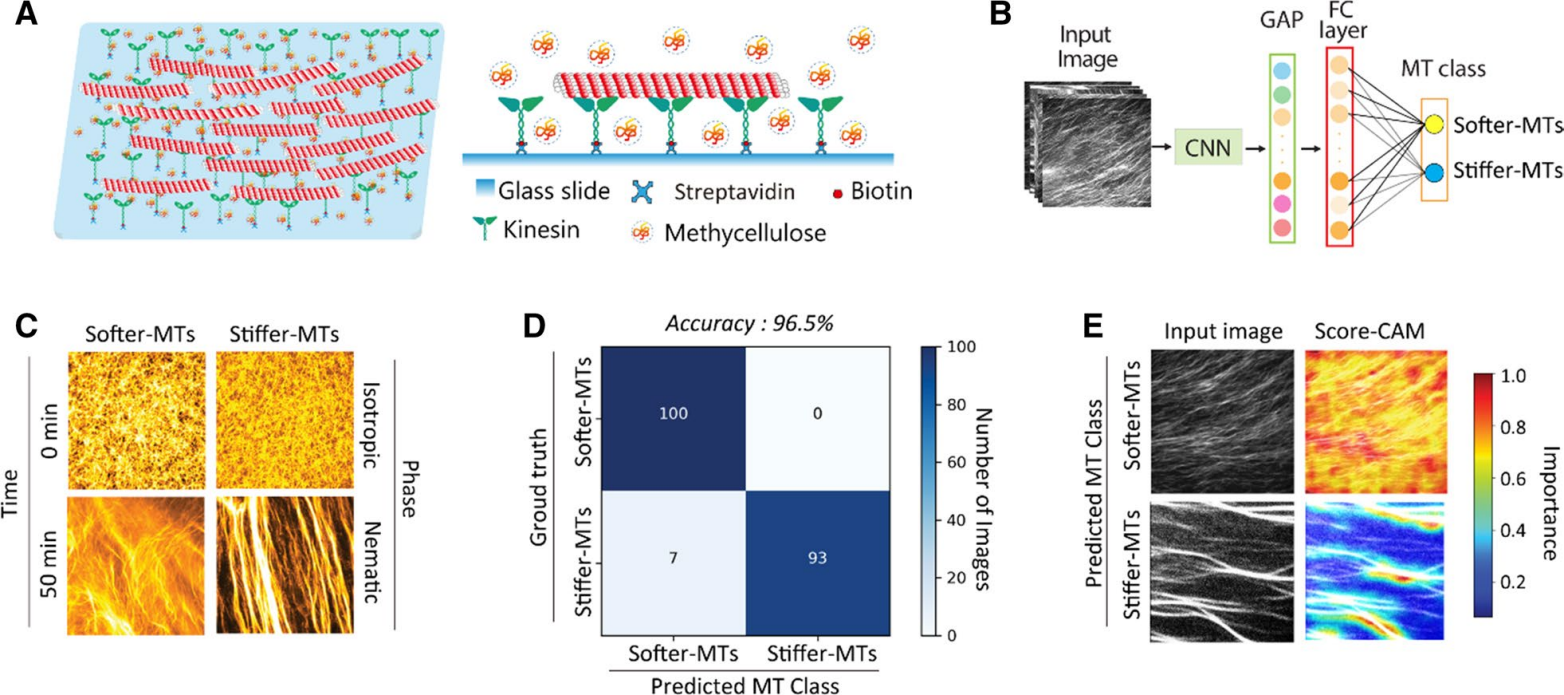
Distinct patterns formed by MTs with different flexural rigidities. **A** MT gliding assays conducted within a reconstructed MT-kinesin system in the presence of methylcellulose. **B** A deep learning CNN model, including global average pooling (GAP) layer, final connected (FC) layer, and MT class output, is constructed to classify the two MTs: *softer-MTs* and *stiffer-MTs*. The two groups of MTs are polymerized at different tubulin concentration of 30 μM and 100 μM, which are named as *softer-MTs* (*κ* = 0.27 × 10^−23^ N m^2^) and *stiffer-MTs* (*κ* = 0.80 × 10^−23^ N m^2^), respectively. **C** From the identical isotropic state, *softer-MTs* and *stiffer-MTs* form distinctive patterns at the nematic phase. The addition time of ATP was set as 0 min. Scale bar = 50 μm. **D** Confusion matrix based on the trained CNN classifier. Here, 200 MT pattern images are categorized using the classifier. **E** The classification strategy of CNN classifier is visually explained using Score-CAM

Distinct patterns of collective motion emerged in the two groups of MTs during the gliding assays. Initially, MTs in both groups were uniformly distributed with random orientations, which could be treated as an isotropic phase (0 min, Fig. 5C). Next, the gliding MTs continually collided with their neighboring MTs in the presence of a depletion force induced by methylcellulose. The gliding directions and orientations of the MTs were tuned by frequent collisions. The collective motion gradually arose in both groups and formed stable nematic patterns after 50 min. *Softer MTs* tended to form local steam, whereas *stiffer MTs* formed stable bundles (50 min, Fig. 5C).

MT patterns formed (at 50 min) by the two MT groups were categorized into two classes, *softer-MTs* and *stiffer-MTs*, using the trained deep CNN classifier. The 200 MT pattern images were categorized using the trained CNN classifier with an accuracy of 96.5% (100% accuracy for *softer-MTs* and 93% accuracy for *stiffer-MTs*) (Fig. 5D). The types of MTs could be predicted and sorted into their corresponding classes according to their patterns, which suggested certain general differences in patterns between the two groups.

The differences in patterns between the two groups were revealed by analyzing the classification strategy of the CNN classifier. Score-CAM was introduced to visually explain how the classifier categorized the MT patterns into the two groups (Fig. 5E) [55]. The more important the area or feature in the image for making the final categorization decision by the classifier, the higher the score gained and the stronger the responses in the CAM. For the images sorted into *softer-MT* groups, a uniformly high activation responded and overlaid the entire image area, which indicated that a large-scale local stream with a relatively even distribution was detected (Fig. 5E). In contrast, for the images sorted into the *stiffer-MT* group, only the bundle area was highly activated in the heatmap, which indicated that a distinguished uneven structure (MT bundle) was identified (Fig. 5E). The differences in the Score-CAM patterns between the two groups were in accordance with our observed results.

The collective motion of MTs has been proposed to emerge due to a depletion force between filaments, which reduces filament crossover, promotes alignment, and leads to a transition of filaments from an active, isotropic state to locally aligned polar patterns [64]. According to the Vicsek model, identical objects, including particles or filaments moving at a constant speed, tend to interact locally by aligning with neighbors [65]. A similar opinion was proposed that self-propelled objects, such as gilding MTs, show progressively larger probabilities to align with their neighbors [66]. Recently, an important supplement to these hypotheses stated that MTs align and are actively transferred from their original bundle to another anti-aligned bundle [67]. All the theories indicated that alignment is a critical step in the formation of collective motion, and the formation of MT bundles is a dynamic process. As an apparent and key factor affecting the gliding and alignment behavior of the filaments, MT flexural rigidity should directly influence the phase transition process of collective motion.

The two groups of MTs enable us to infer that MTs with different *κ* perform differently on the occasions of collision, alignment, and diffusive separation. Compared with the *stiffer-MTs*, the *softer-MTs* are more flexible in changing their gliding directions. Although the bundles of *softer MTs* were formed by the depletion force, the MTs inside the bundle could be easily dispersed in the next collision or disturbed by another anti-aligned bundle. In contrast, the bundles formed by *stiffer-MTs* are long-standing and tend to merge with neighboring bundles, which results in a relatively large bundle. Moreover, the performance differentials of defined *softer MTs and stiffer-MTs* demonstrated that the method for designing flexural rigidity is feasible.

## Conclusions

In summary, an accurate method of measuring MT flexural rigidity was developed by improving the localization precision up to nanometer scale. Based on this improved methodology, we further discovered that flexural rigidity is directly affected by growth rate and goes through three phases among a wide range of tubulin concentrations (20–200 μM), which revealed a new relationship between dynamics and mechanics of MTs and deepened the understanding of the regulatory mechanism of MT flexural rigidity in vivo. These findings not only offer reasonable explanations for long-standing discrepancies in previously measured flexural rigidities, but also provide a convenient method for the production of MTs with predefined stiffness.

Consequently, as shown with the example of MT collective motion, the proposed method would promote the development of potential nanobiotechnology applications by eliminating methodological inconsistencies among assays and providing a controllable experimental environment. Additionally, owing to the simplicity and ease of manipulation, the method for designing flexural rigidity of MTs by altering tubulin concentrations will be a useful quantitative tool to investigate the biological functions of MT rigidity or study the influences of MT flexural rigidity on their routine behaviors in vitro, including gliding motility assays, collective motion, and other biological activities.

Besides tubulin concentration, the regulations of the dynamics and mechanics of MTs in vivo involve many other factors, including MAPs and post-translational modifications. Our results revealed a new quantitative relation between the growth rate and flexural rigidity of MTs by solely modifying the tubulin concentration. It provides comprehensive and comparable data of growth rate and MT rigidity with that in vivo and can be a datum reference for investigating the regulation mechanism of MT mechanics and the functions of specific MAP in the future.

## Supplementary Information

**Additional file 1: Figure S1.** The fabrication process of Au stripe-patterned substrate. **Figure S2.** Measurement process of MT flexural rigidity. **Figure S3.** Summary of mean values of *κ*_meas_/*κ*_set_. **Figure S4.** Measurement process of MT growth rate. **Figure S5.** The training process and performance evaluation of the CNN classifier. **Table S1.** Summary of reported flexural rigidity for MTs. **Table S2.** Summary of the flexural rigidity and growth rate of MTs polymerized using 20–200 μM tubulin. **Table S3.** *P* values of the Steel–Dwass test for MT growth rate at different tubulin concentrations. **Table S4.** *P* values of the Steel–Dwass test for MT flexural rigidity at different tubulin concentrations.

**Additional file 2: Movie S1.** The fluctuation of MT polymerizing under 20 μM tubulin concentration.

**Additional file 3: Movie S2.** The fluctuation of MT polymerizing under 30 μM tubulin concentration.

**Additional file 4: Movie S3.** The fluctuation of MT polymerizing under 50 μM tubulin concentration.

**Additional file 5: Movie S4.** The fluctuation of MT polymerizing under 100 μM tubulin concentration.
