# Supplementary material for: Growth rate-dependent flexural rigidity of microtubules influences pattern formation in collective motion
Source: J Nanobiotechnology. 2021 Jul 19;19:218. doi: 10.1186/s12951-021-00960-y (PMC8287809; doi:10.1186/s12951-021-00960-y)
Supplement: Supplementary file 1 — Additional file 1: Figure S1. The fabrication process of Au stripe-patterned substrate. Figure S2. Measurement process of MT flexural rigidity. Figure S3. Summary of mean values of κmeas/κset. Figure S4. Measurement process of MT growth rate. Figure S5. The training process and performance evaluation of the CNN classifier. Table S1. Summary of reported flexural rigidity for MTs. Table S2. Summary of the flexural rigidity and growth rate of MTs polymerized using 20–200 μM tubulin. Table S3. P values of the Steel–Dwass test for MT growth rate at different tubulin concentrations. Table S4. P values of the Steel–Dwass test for MT flexural rigidity at different tubulin concentrations. [file 12951_2021_960_MOESM1_ESM.docx]

Supporting Information

**Growth rate-dependent flexural rigidity of microtubules influences**

**pattern formation in collective motion**

*Hang Zhou, Naoto Isozaki, Kazuya Fujimoto, and Ryuji Yokokawa**

*Corresponding author.

Department of Micro Engineering, Kyoto University

Kyoto Daigaku-Katsura, Nishikyo-ku, Kyoto 615-8540, Japan

Tel/Fax: +81-75-383-3680

Email: yokokawa.ryuji.8c@kyoto-u.ac.jp

Contents

[1. Supplementary Figures 1](#_Toc66710343)

[2. Supplementary Tables 6](#_Toc66710344)

[3. Supplementary Movies 10](#_Toc66710345)

[4. Supplementary References 11](#_Toc66710346)

# **Supplementary Figures**


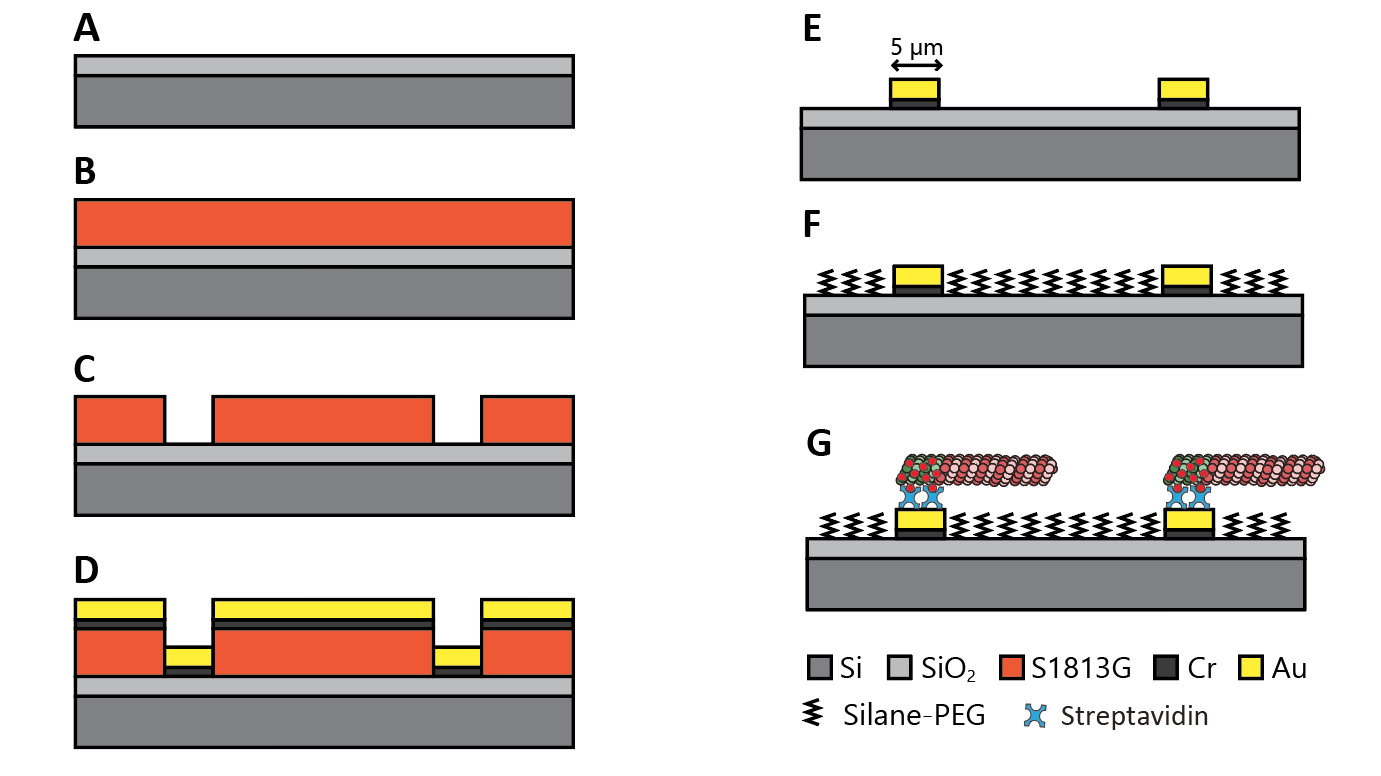


**Figure S1.** The fabrication process of Au stripe-patterned substrate. (A) Si/SiO_2_ substrate was dehydrated at 115 °C for 5 min. (B) Photoresist S1813G was spin-coated as a sacrificial layer with a thickness of 1 µm and (C) developed using CD-26. (D) Cr and Au were successively deposited on S1813-patterned substrates with the thickness of 3 nm and 20 nm, and (E) selectively lifted off with the sacrificial layer by ultrasonicating in acetone. (F) The silane-PEG-SAM layer was coated on SiO_2_ substrates to allow molecules to attach only on Au. (G) Partially biotinylated MTs were immobilized onto the Au-stripe via biotin–streptavidin bindings.


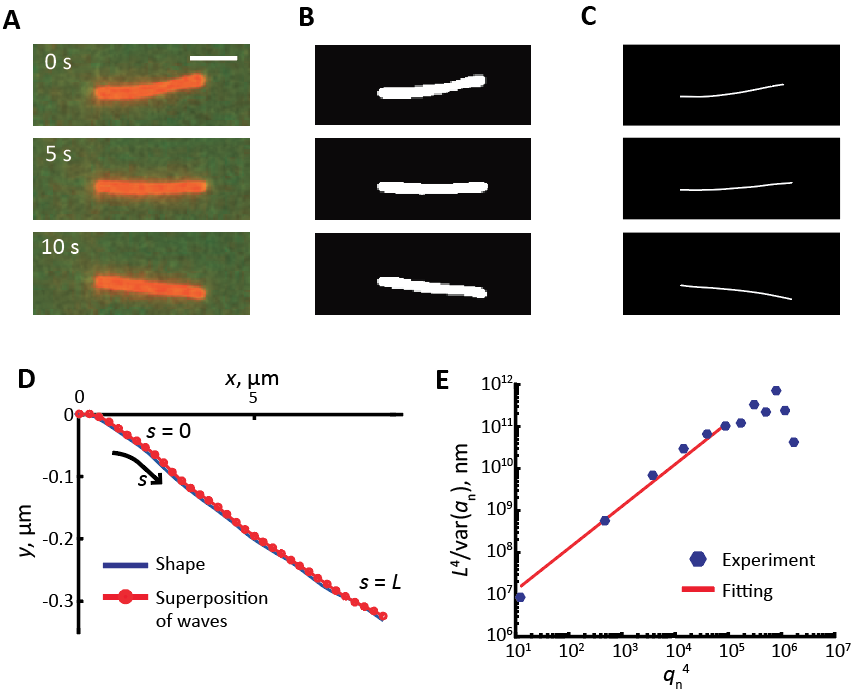


**Figure** **S2.** Measurement process of MT flexural rigidity. (A) Sequential images of a fluctuating MT (red) immobilized on the Au-stripe. Scale bar = 5 μm. Images were (B) binarized and (C) skeletonized. (D) Example of Fourier decomposition of the MT shape for a single frame. Blue line: MT shape. Red line and circles: superposition of waves. (E) Relationship between *q_n_*^4^ and *L^4^*/var(*a_n_*). Blue dots: experimental results. Red line: fitting result.


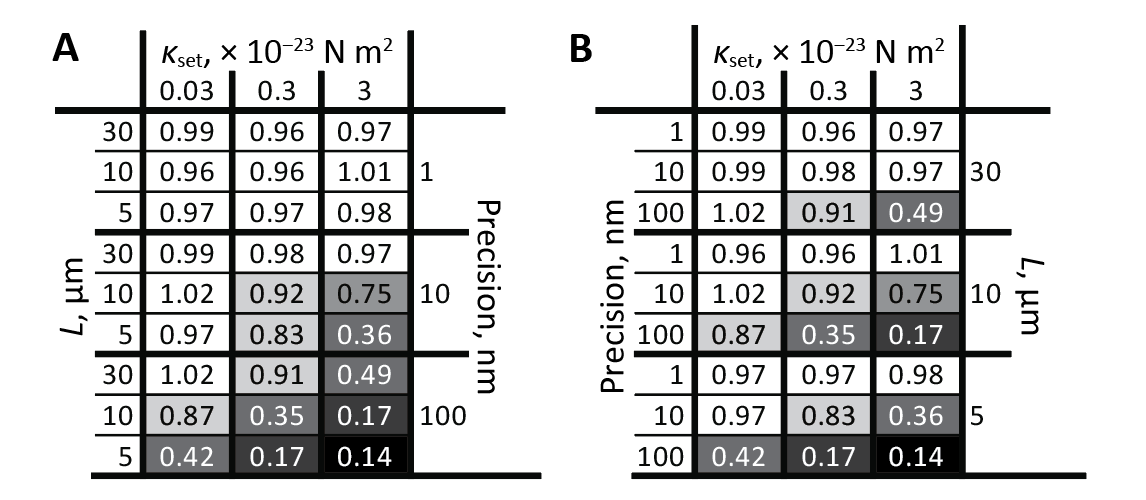


**Figure** **S3.** Summary of mean values of *κ*_meas_/*κ*_set_. The color intensity in a frame corresponds to the degree of error in measurement. (A) Localization precision and *κ*_set_, and (B) *L* and *κ*_set_ are constant in a thick frame.

**
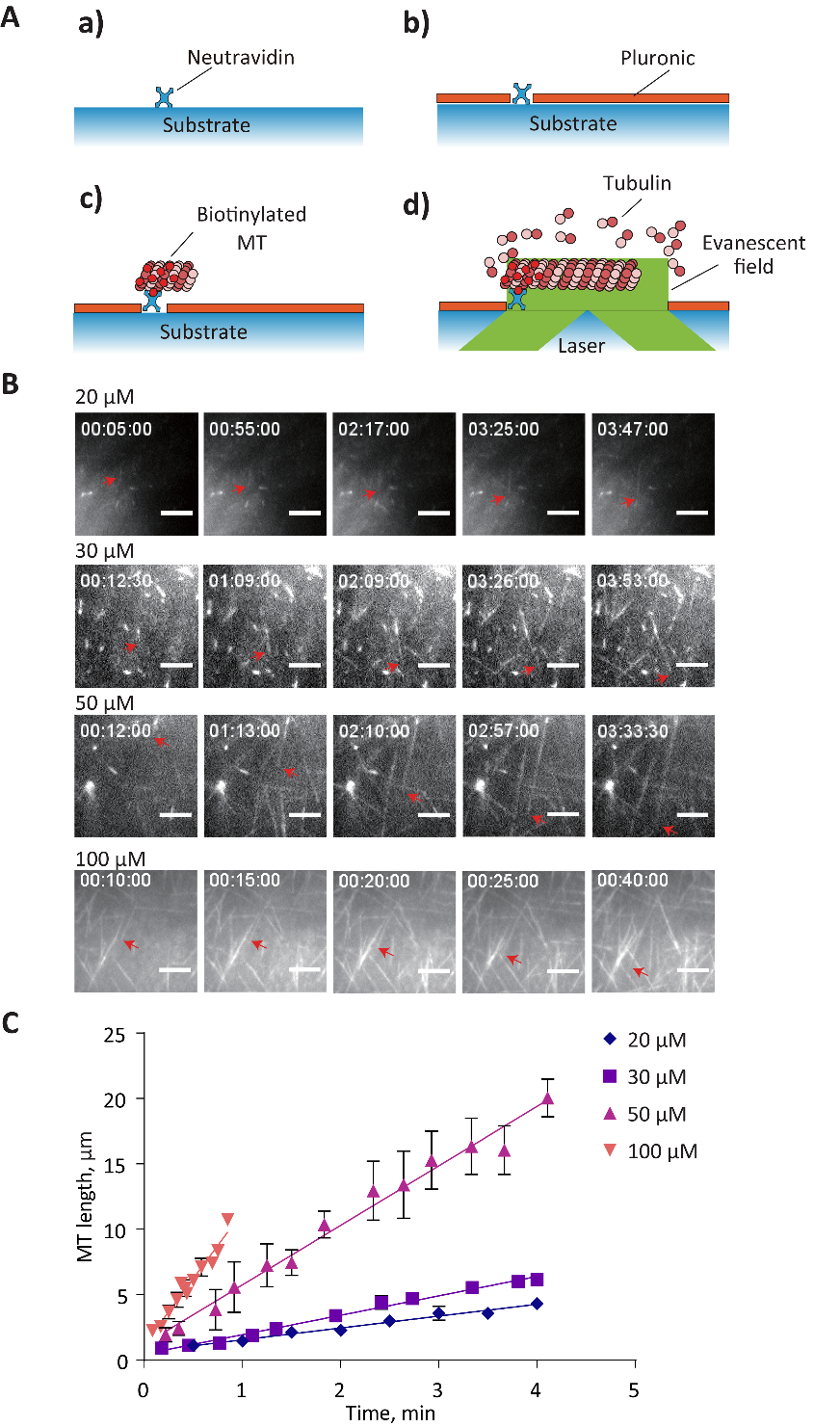
**

**Figure S4.** Measurement process of MT growth rate. (A) Illustration of direct observation for the elongation process of individual growing MTs using TIRF microscopy (from the side view of the bottom coverslip). a) Neutravidin was introduced onto the substrate. After washing the channel thoroughly with BRB80, b) Pluronic solution was introduced into the flow cell for 5 min and washed with BRB80. Thereafter, c) the partially biotinylated seed MTs were introduced and selectively immobilized onto substrates via biotin–neutravidin binding. d) Tubulin protein solution with specific concentration was introduced into the flow cell and MT elongation process was observed using TIRF microscopy. (B) Sequential images of the growing MTs elongating under 20, 30, 50, and 100 µM tubulin concentration. Scale bar = 5 μm. (C) Time course of MT elongation length in the presence of 20, 30, 50, and 100 µM tubulin concentrations. Solid lines: linear fitting by the least squares regression function with the R2 of 0.98, 0.99, 0.98, and 0.96, respectively.

**
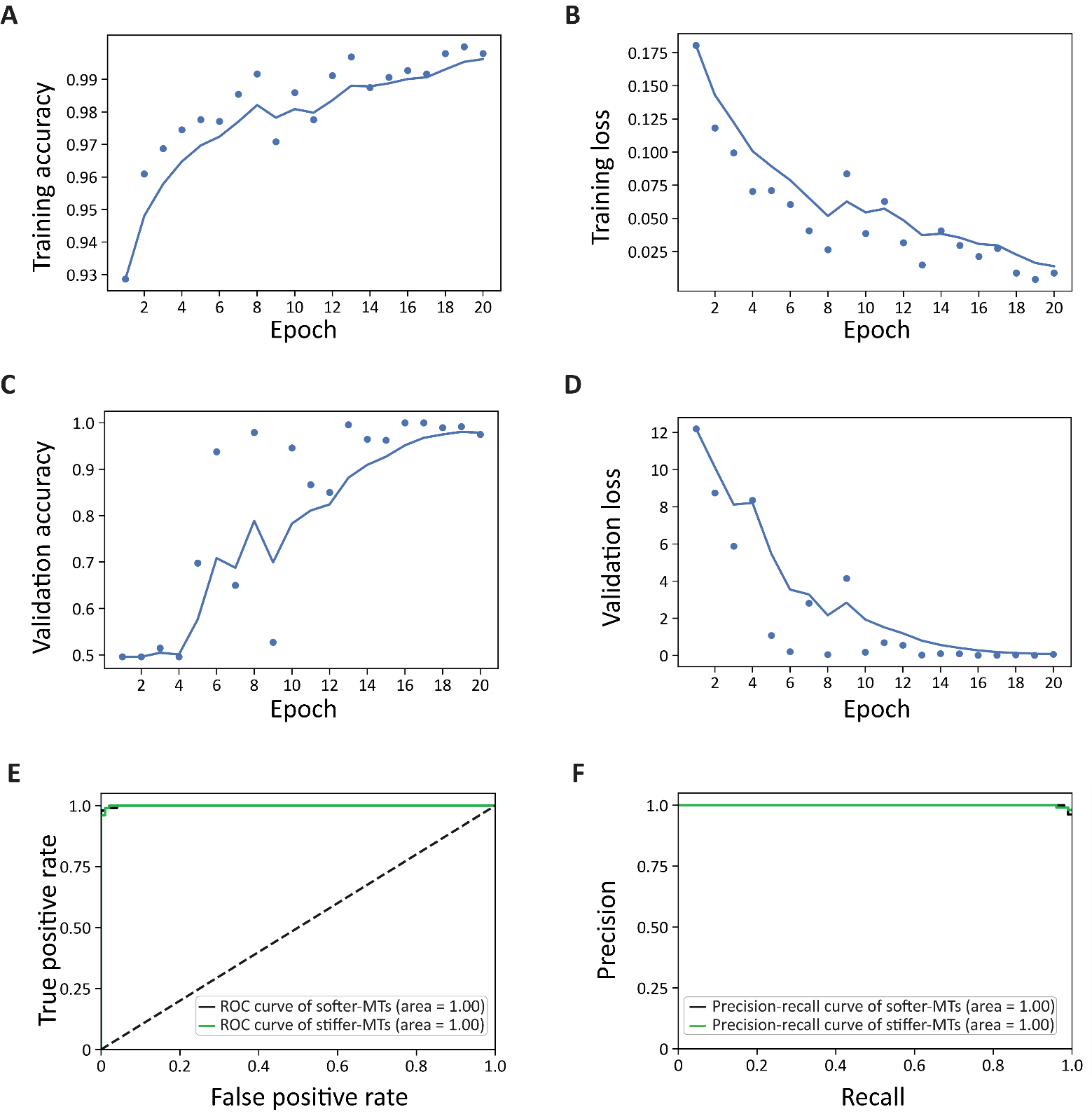
**

**Figure S5.** The training process and performance evaluation of the CNN classifier. (A) The training accuracy was improved from 0.93 to 0.99 with (B) the training loss decreasing from 0.18 to 0.009 in 20 epochs. (C) The validation accuracy was optimized from 0.50 to 0.98 with (D) the validation loss decreasing from 12.20 to 0.059 in 20 epochs. Exponential moving averages with a smoothing factor of 2 (blue lines in (A)–(D)) are used to track the trend of the training and validation process. The performance of the CNN classifier was evaluated by (E) the receiver operating characteristic (ROC) curve, and (F) precision-recall curve. In the classification tasks of softer-MTs and stiffer-MTs, the trained classifier achieved the best performance (with the recall rate of 1.00, and the precision of 1.00) to categorize the two MT groups.

# **Supplementary Tables**

**Table S1. Summary of reported flexural rigidity for MTs**

| **Citation** | **Nucleotide** | **Tubulin conc.** | **Condition** | ***κ*, × 10^−23^**  **[N·m^2^]** | **Method** | **Localization Precision** |
| --- | --- | --- | --- | --- | --- | --- |
| Van den Heuvel *et al.* (2008) [1] | GTP | 36.4 μm | 100 μm tx | 0.1 ± 0.01 | MT trajectory | - |
| Mizushima-Sugano *et al.* (1983) [2] | GTP | 18.2 μm | - | 0.045 | MT shape | - |
| Dye *et al.* (1993) [3] | GTP | 18.2 μm | - | 0.136 | Flow field | - |
|  |  |  | 10 or 50 μm tx | 0.013 |  |  |
|  |  | 7.27 μm | 0.1 mg/ml MAP mix | 0.03 |  |  |
| Mickey and Howard (1995) [4] | GMPCPP | 20 μm | - | 6.2 ± 0.9 | Thermal fluctuation | Pixel |
|  | GTP |  | 10 μm tau | 3.4 ± 0.3 |  |  |
|  |  |  | 10 μm tx | 3.2 ± 0.2 |  |  |
|  |  |  | - | 2.6 ± 0.2 |  |  |
| Felgner *et al.* (1997) [5] | GTP | 9.09 μm | 10 μm tx | 0.10 ± 0.03 | Optical trapping | - |
|  |  |  | - | 0.38 ± 0.09 |  |  |
|  |  |  | 2% tau | 0.45 ± 0.15 |  |  |
|  |  |  | 18% tau | 0.89 ± 0.13 |  |  |
|  |  |  | 48% tau | 0.94 ± 0.23 |  |  |
|  |  |  | 85% tau | 1.04 ± 0.31 |  |  |
| Cassimeris *et al.* (2001) [6] | GTP | 10 μm | - | 1.85 ± 0.20 | Thermal fluctuation | Pixel |
|  |  |  | 0.2 μm XMAP 215 (co) | 1.75 ± 0.22 |  |  |
| Kawaguchi *et al.* (2010) [7] | GMPCPP | 20 µm | 20°C | 1.19 ± 0.11 | Thermal fluctuation | Pixel |
|  |  |  | 35°C | 0.89 ± 0.18 |  |  |
|  | GTP |  | 20°C | 0.81 ± 0.08 |  |  |
|  |  |  | 35°C | 0.48 ± 0.08 |  |  |
|  |  |  | 10 µm tx, 20–35°C | 0.25 ± 0.05 |  |  |
| Hawkins *et al.* (2013) [8] | GTP | 45.5 µm | 50 μm tx | 0.25 ± 0.05 | Thermal fluctuation | Pixel |
|  | GMPCPP |  | - | 0.8 ± 0.2 |  |  |
|  |  |  | 50 μm tx | 0.8 ± 0.3 |  |  |
|  | GTP-γ-S |  | 5 μm tx (co) | 0.21 ± 0.06 |  |  |
|  |  |  | 50 μm tx | 0.21 ± 0.05 |  |  |
|  | GTP |  | 50 μm tx, 1:100 tau (co), 1:1 tau | 1.5 ± 0.4 |  |  |
|  |  |  | 50 μm tx, 1:1 tau | 0.2 ± 0.03 |  |  |
|  |  |  | 50 μm tx, 1:2 MAP4 | 0.22 ± 0.04 |  |  |
| Gittes *et al.* (1993) [9] | GTP | 18.5–54.5 µm | 10 µm tx | 2.2 ± 0.2 | Thermal fluctuation | Pixel |
|  |  |  | 10 µm tx, non-labeled | 2.1 ± 0.1 |  |  |
| Felgner *et al.* (1996) [10] | GTP | 9.09 µm | 10 µm tx | 0.10 ± 0.03 | Optical trapping | Pixel |
|  |  |  | - | 0.37 ± 0.08 |  |  |
|  |  |  | MAPs | 1.6 ± 0.3 |  |  |
| Venier *et al.* (1994) [11] | GTP | 12 µm | 50 µm tx (co), 10 µm tx | 0.47 ± 0.04 | Thermal fluctuation | Pixel |
|  |  | 20 µm | - | 0.92 ± 0.09 |  |  |
|  |  |  |  | 0.85 ± 0.2 | Flow field |  |
| Janson and Dogterom (2004) [12] | GTP | 20 µm | O_2_-scavenger (co) | 2.15 ± 0.79 | Thermal fluctuation | Pixel |
|  |  | 28 µm | - | 1.41 ± 0.66 |  |  |
| Janson and Dogterom (2004) [13] | GTP | 28 µm | O_2_-scavenger (co) | 1.74 ± 0.33 | Thermal fluctuation | Pixel |
|  |  | 26 µm | - | 2.73 ± 0.87 |  |  |
| Dogterom and Yurke (1997) [14] | GTP | 25 µm | O_2_-scavenger (co) | 3.44 ± 0.70 | Thermal fluctuation | Pixel |
| Valdman *et al.* (2012) [15] | GTP | 20 µm | 1, 6.5, 20 μm tx (co),  10 μm tx | 1.43 ± 0.22 | Thermal fluctuation | ~ 0.1 pixel |
| Lopez *et al.* (2014) [16] | GTP | 50 µm | 1, 7, 20 μm tx (co) | 0.99 | Thermal fluctuation | ~ 0.1 pixel |
|  | GMPCPP |  | - | 2.82 |  |  |
|  |  |  | 1, 7, 20 μm tx (co) | 1.99 |  |  |
|  |  |  | 200 nm EB1 | 5.42 |  |  |
|  | GTP-γ-S |  | 1, 7, 20 μm tx (co) | 1.32 |  |  |
|  |  |  | 1, 7, 20 µm tx (co),  500 nm EB1 | 2.07 |  |  |

* Paclitaxel is abbreviated as tx; (co) in condition represents the situations in which MTs were copolymerized in the presence of reagents.

**Table S2. Summary of the flexural rigidity and growth rate of MTs polymerized using 20–200 μM tubulin**

| **Tubulin conc.** | **20 μM** | **30 μM** | **35 μM** | **40 μM** | **50 μM** | **75 μM** | **100 μM** | **150 μM** | **200 μM** |
| --- | --- | --- | --- | --- | --- | --- | --- | --- | --- |
| **Flexural rigidity,**  **× 10^−23^ N**·**m^2^** | 0.71 ± 0.36 | 0.80 ± 0.34 | 0.75 ± 0.29 | 0.70 ± 0.32 | 0.63 ± 0.20 | 0.43 ± 0.31 | 0.27 ± 0.10 | 0.31 ± 0.18 | 0.32 ± 0.39 |
| **Growth rate,**  **μm**·**min^−1^** | 0.92 ± 0.10 | 1.37 ± 0.07 | 2.37 ± 0.08 | 2.81 ± 0.20 | 4.09 ± 0.56 | 8.05 ± 0.30 | 10.10 ± 0.46 | 14.08 ± 0.51 | 16.31 ± 1.08 |

* Data of flexural rigidity are shown as mean ± S. D. for *N* = 14, 9, 10, 6, 20, 8, 12, 13, and 6, respectively. Growth rate data are shown as mean ± S. E. for *N* = 7, 10, 20, 15, 10, 11, 9, 12, and 10, respectively.

**Table S3. *P* values of the Steel–Dwass test for MT growth rate at different tubulin concentrations**

| **Tubulin conc.** | **Tubulin conc.** | | | | | | | | |
| --- | --- | --- | --- | --- | --- | --- | --- | --- | --- |
|  | **20 μM** | **30 μM** | **35 μM** | **40 μM** | **50 μM** | **75 μM** | **100 μM** | **150 μM** | **200 μM** |
| **20 μM** |  | *p >* 0.05 | *p* < 0.05 | *p* < 0.05 | *p* < 0.05 | *p* < 0.05 | *p* < 0.05 | *p* < 0.05 | *p* < 0.05 |
| **30 μM** | *p >* 0.05 |  | *p* < 0.05 | *p* < 0.05 | *p* < 0.05 | *p* < 0.05 | *p* < 0.05 | *p* < 0.05 | *p* < 0.05 |
| **35 μM** | *p* < 0.05 | *p* < 0.05 |  | *p >* 0.05 | *p >* 0.05 | *p* < 0.05 | *p* < 0.05 | *p* < 0.05 | *p* < 0.05 |
| **40 μM** | *p* < 0.05 | *p* < 0.05 | *p >* 0.05 |  | *p >* 0.05 | *p* < 0.05 | *p* < 0.05 | *p* < 0.05 | *p* < 0.05 |
| **50 μM** | *p* < 0.05 | *p* < 0.05 | *p >* 0.05 | *p >* 0.05 |  | *p* < 0.05 | *p* < 0.05 | *p* < 0.05 | *p* < 0.05 |
| **75 μM** | *p* < 0.05 | *p* < 0.05 | *p* < 0.05 | *p* < 0.05 | *p* < 0.05 |  | *p >* 0.05 | *p* < 0.05 | *p* < 0.05 |
| **100 μM** | *p* < 0.05 | *p* < 0.05 | *p* < 0.05 | *p* < 0.05 | *p* < 0.05 | *p >* 0.05 |  | *p* < 0.05 | *p* < 0.05 |
| **150 μM** | *p* < 0.05 | *p* < 0.05 | *p* < 0.05 | *p* < 0.05 | *p* < 0.05 | *p* < 0.05 | *p* < 0.05 |  | *p >* 0.05 |
| **200 μM** | *p* < 0.05 | *p* < 0.05 | *p* < 0.05 | *p* < 0.05 | *p* < 0.05 | *p* < 0.05 | *p* < 0.05 | *p >* 0.05 |  |

**Table S4. *P* values of the Steel–Dwass test for MT flexural rigidity at different tubulin concentrations**

| **Tubulin conc.** | **Tubulin conc.** | | | | | | | | |
| --- | --- | --- | --- | --- | --- | --- | --- | --- | --- |
|  | **20 μM** | **30 μM** | **35 μM** | **40 μM** | **50 μM** | **75 μM** | **100 μM** | **150 μM** | **200 μM** |
| **20 μM** |  | *p >* 0.05 | *p >* 0.05 | *p >* 0.05 | *p >* 0.05 | *p >* 0.05 | *p* < 0.05 | *p >* 0.05 | *p >* 0.05 |
| **30 μM** | *p >* 0.05 |  | *p >* 0.05 | *p >* 0.05 | *p >* 0.05 | *p >* 0.05 | *p >* 0.05 | *p >* 0.05 | *p >* 0.05 |
| **35 μM** | *p >* 0.05 | *p >* 0.05 |  | *p >* 0.05 | *p >* 0.05 | *p >* 0.05 | *p* < 0.05 | *p >* 0.05 | *p >* 0.05 |
| **40 μM** | *p >* 0.05 | *p >* 0.05 | *p >* 0.05 |  | *p >* 0.05 | *p >* 0.05 | *p >* 0.05 | *p >* 0.05 | *p >* 0.05 |
| **50 μM** | *p >* 0.05 | *p >* 0.05 | *p >* 0.05 | *p >* 0.05 |  | *p >* 0.05 | *p* < 0.05 | *p* < 0.05 | *p >* 0.05 |
| **75 μM** | *p >* 0.05 | *p >* 0.05 | *p >* 0.05 | *p >* 0.05 | *p >* 0.05 |  | *p >* 0.05 | *p >* 0.05 | *p >* 0.05 |
| **100 μM** | *p* < 0.05 | *p >* 0.05 | *p* < 0.05 | *p >* 0.05 | *p* < 0.05 | *p >* 0.05 |  | *p >* 0.05 | *p >* 0.05 |
| **150 μM** | *p >* 0.05 | *p >* 0.05 | *p >* 0.05 | *p >* 0.05 | *p* < 0.05 | *p >* 0.05 | *p >* 0.05 |  | *p >* 0.05 |
| **200 μM** | *p >* 0.05 | *p >* 0.05 | *p >* 0.05 | *p >* 0.05 | *p >* 0.05 | *p >* 0.05 | *p >* 0.05 | *p >* 0.05 |  |

# **Supplementary Movies**

**Movie S1.** The fluctuation of MT polymerizing under 20 μM tubulin concentration. An MT, which polymerized from 20 μM tubulin solution, was tethered at one end with its right segment (orange red) fluctuating freely under thermal driving. Images were taken at 2.5 frames s^-1^. The playback rate is 15 × real-time (see the timestamp). Scale bar = 5 μm.

**Movie S2.** The fluctuation of MT polymerizing under 30 μM tubulin concentration. An MT, which polymerized from 30 μM tubulin solution, was tethered at one end with its right segment (orange red) fluctuating freely under thermal driving. Images were taken at 2.5 frames s^-1^. The playback rate is 15 × real-time (see the timestamp). Scale bar = 5 μm.

**Movie S3.** The fluctuation of MT polymerizing under 50 μM tubulin concentration. An MT, which polymerized from 50 μM tubulin solution, was tethered at one end with its right segment (orange red) fluctuating freely under thermal driving. Images were taken at 2.5 frames s^-1^. The playback rate is 15 × real-time (see the timestamp). Scale bar = 5 μm.

**Movie S4.** The fluctuation of MT polymerizing under 100 μM tubulin concentration. An MT, which polymerized from 100 μM tubulin solution, was tethered at one end with its right segment (orange red) fluctuating freely under thermal driving. Images were taken at 2.5 frames s^-1^. The playback rate is 15 × real-time (see the timestamp). Scale bar = 5 μm.

# **Supplementary References**

1. Heuvel MGLV den, Graaff MP de, Dekker C. Microtubule curvatures under perpendicular electric forces reveal a low persistence length. Proc. Natl. Acad. Sci. U.S.A. 2008;105:7941–6.
2. Mizushima-Sugano J, Maeda T, Miki-Noumura T. Flexural rigidity of singlet microtubules estimated from statistical analysis of their contour lengths and end-to-end distances. Biochim. Biophys. Acta BBA - Gen. Subj. 1983;755:257–62.
3. Dye RB, Fink SP, Williams RC. Taxol-induced flexibility of microtubules and its reversal by MAP-2 and Tau. J Biol Chem. 1993;268:6847–50.
4. Mickey B, Howard J. Rigidity of microtubules is increased by stabilizing agents. J. Cell Biol. 1995;130:909–17.
5. Felgner H, Frank R, Biernat J, Mandelkow E-M, Mandelkow E, Ludin B . Domains of Neuronal Microtubule-associated Proteins and Flexural Rigidity of Microtubules. J Cell Biol. 1997;138:1067–75.
6. Cassimeris L, Gard D, Tran PT, Erickson HP. XMAP215 is a long thin molecule that does not increase microtubule stiffness. J. Cell Sci. 2001;114:3025–33.
7. Kawaguchi K, Yamaguchi A. Temperature dependence rigidity of non-taxol stabilized single microtubules. Biochem. Biophys. Res. Commun. 2010;402:66–9.
8. Hawkins TL, Sept D, Mogessie B, Straube A, Ross JL. Mechanical Properties of Doubly Stabilized Microtubule Filaments. Biophys. J. 2013;104:1517–28.
9. Gittes F. Flexural rigidity of microtubules and actin filaments measured from thermal fluctuations in shape. J Cell Biol. 1993;120:923–34.
10. Felgner H, Frank R, Schliwa M. Flexural rigidity of microtubules measured with the use of optical tweezers. J Cell Sci.1996;109:509–16.
11. Venier P, Maggs AC, Carlier MF, Pantaloni D. Analysis of microtubule rigidity using hydrodynamic flow and thermal fluctuations. J Biol Chem. 1994;269:13353–60.
12. Janson ME, Dogterom M. Scaling of Microtubule Force-Velocity Curves Obtained at Different Tubulin Concentrations. Phys Rev Lett. 2004;92:248101.
13. Janson ME, Dogterom M. A bending mode analysis for growing microtubules: evidence for a velocity-dependent rigidity. Biophys J. 2004;87:2723–36.
14. Dogterom M, Yurke B. Measurement of the Force-Velocity Relation for Growing Microtubules. Science. 1997;278:856–60.
15. Valdman D, Atzberger PJ, Yu D, Kuei S, Valentine MT. Spectral Analysis Methods for the Robust Measurement of the Flexural Rigidity of Biopolymers. Biophys J. 2012;102:1144–53.
16. Lopez BJ, Valentine MT. Mechanical effects of EB1 on microtubules depend on GTP hydrolysis state and presence of paclitaxel: Effects of EB1 on Microtubule Mechanics. Cytoskeleton. 2014;71:530–41.
